# The collaborative framework for the management of tuberculosis and type 2 diabetes syndemic in low- and middle-income countries: a rapid review

**DOI:** 10.1186/s12889-024-18256-9

**Published:** 2024-03-07

**Authors:** Denise Michela Milice, Ivalda Macicame, José L.Peñalvo

**Affiliations:** 1 Instituto Nacional de Saúde, Maputo, Mozambique; 2 Global Health Institute, University of Antwerp, Wilrijk, Belgium; 3 National Center for Epidemiology, Instituto de Salud Carlos III, Madrid, Spain

**Keywords:** framework, Screening, Co-management, Type 2 diabetes, Tuberculosis, Low- and middle-income countries

## Abstract

**Introduction:**

Given the absence of international guidelines on the joint management and control of tuberculosis (TB) and type 2 diabetes mellitus (T2D), the World Health Organization (WHO) and the International Union Against Tuberculosis and Lung Disease (The Union) launched in 2011 a policy framework to address the growing syndemic burden of TB-T2D. This review aimed at mapping the available evidence on the implementation of the Union-WHO Framework, explicitly, or bi-directional TB-T2D health programs as an initiative for co-management in patients in low- and middle-income countries (LMIC).

**Methods:**

A rapid review was performed based on a systematic search in PubMed and Web of Science electronic databases for peer-reviewed articles on The Union-WHO Framework and bi-directional interventions of TB and T2D in LMIC. The search was restricted to English language articles and from 01/08/2011 to 20/05/2022.

**Results:**

A total of 24 articles from 16 LMIC met the inclusion criteria. Four described the implementation of The Union-WHO Framework and 20 on the bi-directional interventions of TB and T2D. Bi-directional activities were found valuable, feasible and effective following the Union-WHO recommendations. Limited knowledge and awareness on TB-T2D comorbidity was identified as one of the barriers to ensure a functional and effective integration of services.

**Conclusions:**

This review revealed that it is valuable, feasible and effective to implement bi-directional TB and T2D activities (screening and management) according to the Union-WHO Framework recommendations, especially in countries that face TB-T2D syndemic. Additionally, it was apparent that gaps still exist in research aimed at providing evidence of costs to implement collaborative activities. There is need for TB and T2D services integration that should be done through the well-stablished TB programme. This integration of two vertical programmes, could ensure patient-centeredness, continuum of care and ultimately contribute for health systems strengthening.

**Supplementary Information:**

The online version contains supplementary material available at 10.1186/s12889-024-18256-9.

## Background

The coexisting burden of tuberculosis (TB) and diabetes mellitus type 2 (T2D) has become an important global public health concern [1]. According to the World Health Organization (WHO) global TB report 2023, there were 7.5 million people newly diagnosed and 1.30 million deaths resulting from TB [2]. In 2021, there were 537 million cases and 6.7 million deaths due to diabetes (90–95% T2D) were registered [3]. The synergies between TB infection and T2D have been known for years, though most of the research carried out in developed countries because in the past, non-communicable diseases (NCDs) such as T2D have not been considered of public health relevance in developing countries [4]. This is changing rapidly with T2D being already a major and increasing public health threat in low- and middle-income countries (LMIC) [1]. This increasing trend is driven mainly by demographic and epidemiological transitions that also accelerate the nutritional (lifestyle) transitions, leading to increased rates of obesity and T2D [5]. The increasing numbers of T2D in LMIC coincides with still much prevalent TB, in a situation that can be defined as syndemic. The term syndemic refers to synergistic health problems that affect the health of a population within the context of persistent and economic inequalities [4]. It was developed by a medical anthropologist Merrill Singer in the early 1990s to call attention to the synergistic nature of the health and social problems facing the poor and underserved [6]. Syndemic is used when communities experience co-occurring epidemics that additively increase negative health consequences [7].

According to the most recent data from the World Bank low-income countries represent 9% of the world population while 76% of the world population were classified as middle-income countries [8]. Thus, LMIC host 85% of the world’s population and bear a disproportionate burden of about 95% of TB cases and 75% of people living with T2D [9, 10].

A meta-analysis conducted in 2018 on the prevalence of TB in T2D patients in African and Asian countries found a pooled prevalence of 4.72% (95% CI: 3.62–5.83) [11]. A systematic review conducted in 2019 on the co-existence of T2D and TB in LMIC reported a wide range among the studies reviewed from 1.8% to 45% of TB patients also reporting T2D [12].

Based on earlier published summary effect estimates, T2D increases the risk of active TB by 3.11–fold and latent TB by 1.18–fold [12, 13]. Conversely, TB can constitute a risk factor for T2D temporarily causing impaired glucose tolerance and predisposing patients to T2D [14].

Given the relevance of the issue and the absence of international guidelines on the co-management and control of both TB and T2D, the WHO and the International Union Against Tuberculosis and Lung Disease (The Union) launched in 2011 a policy framework to address the growing TB-T2D syndemic, known as the collaborative framework for care and control of TB and T2D (The Union-WHO Framework) [15, 16]. This framework aims to guide national programmes on the prevention and control of T2D and TB on how to establish a coordinated response to both diseases, and clinicians on integrated TB-T2D care. It is also intended to help stimulate operational research. As more scientific evidence is documented from the countries experience, this framework should be further developed into global policy and guidelines for collaborative activities. The Union-WHO Framework recommends three important intervention strategies[17]., namely, 1) establishing mechanisms of collaboration between TB and T2D control programmes, 2) detection and management of TB in patients with T2D, and 3) detection and management of T2D in TB patients.

The rational for The Union-WHO Framework takes in consideration that in all high TB burden countries there is an established national TB control programme. It also takes as example of feasibility, the best practices of the collaboration between TB and HIV/AIDS programmes during the last decades that has helped avoid unnecessary duplication of service delivery structures by installing for example, one-stop consultations and overall improving coordination of activities in resource-scarce health-care services [15, 17].

A previous scoping review [17] of the 10 years of implementation of The Union-WHO Framework, at global level, demonstrated that although gaps still exist in research aimed at providing evidence of improved techniques for detecting TB-T2D comorbidity, bi-directional screening is feasible and can potentially improve the diagnosis and co-management of individuals with TB and T2D [16].

The purpose of this review is to map the available evidence on the implementation of the collaborative framework and bi-directional TB-T2D health services as an initiative for co-management in patients in LMIC. This review will add to the existing knowledge by mapping the evidence in low-resource settings also facing the consequences of demographic and epidemiological transitions and the coexistence of the syndemic burden of both communicable and NCDs.

## Methods

### Study design

We were guided by rapid review methods, which is a simplified approach to systematic review approach for synthesizing evidence, to produce findings within a short time.

This review mapped literature related to the implementation of The Union-WHO Framework, explicitly, as well as bi-directional strategies for the management of TB-T2D [18]. A systematic search was conducted to synthesise published literature studies to answer the research question considering qualitative, quantitative and mixed methods. The methodology for this rapid review was based on Preferred Reporting Items for Systematic Reviews and Meta-Analysis (PRISMA) guidelines extension for Scoping Reviews [19] and guided by Arskey and O’Malley’s framework [20] that presents five steps to be used for exploring core concepts and identifying the existing evidence in the research area, namely: (1) identifying the research question, (2) identifying relevant studies, (3) study selection, (4) charting the data, (5) collating, summarizing and reporting the results.

### Identifying the research question

To develop the review question, the PICo approach (Population, Phenomenon of Interest and Context) was used [21]. Based on the approach, the study population was patients with TB and patients with T2D, the phenomenon of interest The Union-WHO Framework and the context was LMIC. The main research question: What evidence is available regarding the extent of implementation and results of the collaborative framework and bi-directional activities for the efficient management of TB and T2D amongst patients with both diseases in LMIC, in order to address gaps and challenges in the TB-T2D continuous and integrated care?

### Outcomes

In this study, feasibility, effectiveness, availability, readiness, and co-management were considered outcomes evaluated according to the report of included studies. Feasibility assessment refers to the extent to which the implementation of TB-T2D screening, diagnosis and management can be successfully employed or carried out in the contexts of the studies evaluated [22].

Value in healthcare is the measured improvement in a patient’s health outcomes for the cost of achieving that improvement [23]. However, the goal of a value-based health care is better health outcomes [24]. In this study we consider effectiveness as the degree to which TB-T2D screening, diagnosis and management are successful in producing the desired results namely, TB patients being diagnosed and managed for T2D [25]. Acceptability is a multi-faceted construct that reflects the extent to which people delivering or receiving a healthcare intervention consider it to be appropriate, based on anticipated or experimental cognitive and emotional responses to the interventions [26].

### Identifying relevant studies

We developed a comprehensive search approach based on PICo terms for published articles relevant to answer our research question. Our search approach included Boolean terms (AND, OR) and Medical Subject Headings terms. The search keywords were: ‘Framework’, ‘Screening’, ‘Co-management’, ‘Diabetes’, ‘Type 2 Diabetes’, ‘Tuberculosis’, ‘Low- and middle-income countries’ and ‘developing countries’ adapted to two databases, namely, PubMed and Web of science. Studies obtained through database searches were exported to Mendeley library for further abstract and full articles screening, respectively. Appropriate MeSH terms and Title/Abstract field tags were used, supported by free-text formats. Some filters were used to limit the research period of 11 years (01/08/2011 to 20/05/2022), given that the Union-WHO Framework was first launched in 2011, and thus capture recent evidence and concepts; articles written in English and exclusively research on human subjects.

### Study selection

All articles were screened by one investigator to test the eligibility criteria by first screening title and abstract. Then, a second screening of the retained studies was performed to select full text articles, for data extraction and analysis.

### Eligibility criteria

The eligibility criteria were developed to ensure the inclusion of specific information related to the search question in the studies. The inclusion criteria in this review comprised studies presenting evidence of TB-T2D comorbidity among patients with TB and T2D. It also considered studies presenting evidence of bi-directional screening and/or management in TB and T2D patients of all ages, as well as studies presenting evidence of implementation of integrated care of TB and T2D. All these studies were divided in two categories, a) studies that clearly stated that the TB-T2D integrated care is aligned with The Union-WHO Framework and b) studies that describe the bi-directional TB-T2D interventions without a clear link with The Union-WHO Framework. This distinction makes possible to have an idea about the number of countries that have formally adopted the framework versus the number of countries implementing co-management even without specific guidelines or links with The Union-WHO Framework. Studies presenting evidence on type 1 diabetes and those focusing exclusively on HIV or co-infections other than TB were excluded. This review only included articles on type 2 diabetes because this type contributes 90–95% of all diabetes cases, worldwide, and shares socio-economic, environmental, and behavioural factors with TB [27, 28].

### Data charting

Data were charted based on a data extraction sheet that was developed, as per rapid review methodologies (Additional file 1). The extraction fields in the sheet were then modified and adapted to fit each category of articles to be included, see Table 1 (information on The Union-WHO Framework) and Table 2 (bi-directional interventions of TB and T2D).

**Table 1:** Information on the Union-WHO framework

Author, Year	Country Rural / Urban Public / Private	Study design	Study population	Findings	Barriers & Facilitators	Outcomes
Ekeke, 2017 [29],	Nigeria Urban Public & private	Cross-sectional	TB patients	T2D prevalence was 9.4%; Factors associated with T2D were: age > 40 years old, rural residence and private health facility care	**Barriers:** Diagnostic method used **Facilitators:** Authors were able to implement the screening in a routine programme setting across multiple regions and facilities with minimal additional costs and training	**Feasibility and effectiveness of T2D screening among TB patients**
Shayo, 2019 [30]	Tanzania Rural & Urban Public & Private	Cross-sectional	Health facilities	Only 38.4% of all T2D facilities offer diagnosis and treatment for TB; The overall readiness of T2D facilities to provide TB services was low (12.6%); Public T2D facilities had comparatively higher availability of TB services than private ones	**Barriers:** Shortage of staff trained to co-manage TB in T2D care facilities; Inadequate TB management guidelines, medications, and diagnostics **Facilitators:** Ministry of Health (MoH) has developed the guideline for TB-T2D collaborative care; NCDs strategic plan II prioritises to train healthcare providers on the collaborative TB-T2D care	**Availability and readiness of TB management in T2D facilities**
Salifu, 2020 [31]	Ghana Public	Exploratory Qualitative	Healthcare workers (HCW)	Implementing bi-directional screening was achievable, when properly implemented; Screening to detect TB among T2D patients was more organised and focused; TB task-shifting officers improved T2D patients screening for TB	**Barriers:** Delays in screening; Fear and stigmatisation of TB; Poor collaboration between TB and T2D units; Skewed funding for screening **Facilitators:** Increase in staff capacity; Institutionalisation of bidirectional screening	**Co-management of TB-T2D comorbidity**
Salifu, 2021 [17], [32]	Ghana Public	Exploratory Qualitative	HCW	The study revealed 3 major themes: (1) Prioritisation of TB/HIV co-infection while negating TB-T2D comorbidity, (2) Poor working conditions, and (3) Coping mechanisms & 5 sub-themes: (1) Low knowledge and awareness on TB-T2D comorbidity, (2) Limited awareness of The Union-WHO framework among the HCW, (3) High workload in TB & T2D clinics, (4) Multiple roles, (5) Inadequate training and (6) Space shortage	**Barriers:** Prioritisation of TB-HIV co-infection while negating TB-T2D comorbidity, Poor working conditions, Low knowledge, and awareness on TB-T2D comorbidity, Limited awareness of The Union-WHO framework among the HCW, High workload in TB & T2D clinics, Multiple roles, Inadequate training, and Space shortage **Facilitators:** Coping mechanisms	**Co-management of TB-T2D comorbidity**

**Table 2:** Bi-directional interventions of TB and T2D

Author, Year	Country Rural / Urban Public / Private	Study design	Study population	Findings	Barriers & Facilitators	Outcomes
Achanta, 2013 [33]	India Rural Public	Cross-sectional	TB patients	TB prevalence was 5.1%; Screening of TB patients for T2D can be effectively implemented within the existing framework of health care delivery; Age was a factor significantly associated with the prevalence of T2D	**Facilitators:** The study was implemented without any additional resources within the existing health care system and with minimum training needs; Screening of patients was well accepted in the community;	**Feasibility of screening for T2D among TB patients**
Dave, 2013 [34]	India	Cross-sectional	TB patients	At 6.5%, the prevalence of T2D in TB patients was low compared with other pilot sites in India; Age ⩾35 years was associated with T2D	**Barriers:** there was no free supply of oral hypoglycaemic drugs, and some patients had to pay for these as out-of-pocket expenses **Facilitators:** Screening was implemented within the routine system with existing staff; With just one day of training, clinical and nursing staff were able to follow the diagnostic algorithm and record appropriate data	**Feasibility of screening for T2D among TB patients**
Prakash, 2013 [35]	India	Cross-sectional	TB patients	The T2D prevalence was 6.3%. A higher prevalence of T2D was found among patients aged ⩾40 years, patients with pulmonary TB and smokers	**Facilitators:** Bi-directional screening for TB and T2D implemented using existing resources and staff, thus indicating that this is feasible; Low loss to follow-up due to the close proximity of the TB and T2D clinics	**Co-management of TB-T2D comorbidity**
Mtwangambate 2014 [36]	Tanzania	Prospective cohort	T2D patients	The prevalence of TB among adults with T2D was sevenfold higher than that reported in the general population	**Barriers:** High rates of non-productive cough **Facilitators:** Low-cost, ‘cough-triggered’ TB case-finding strategy that may serve as a reasonable first step for improving TB screening among adults with T2D in resource-limited settings	**Feasibility of screening for TB among T2D patients**
Viney, 2015	Republic of Kiribati	Case–control	TB cases and controls	The T2D prevalence in cases (101, 37%) was significantly greater than in controls (94, 19%); Screening for T2D in the TB clinic is a worthwhile public health intervention, provided that patients with T2D can access T2D care		**Co-management of TB-T2D comorbidity**
Workne, 2016 [37]	Ethiopia	Exploratory Qualitative	HCW, programme managers, stakeholders	Main themes identified: 1. Unavailability of system for continuity of T2D care; 2. Inadequate knowledge and skills of HCW; 3. Frequent stockouts of T2D supplies; 4. Patient’s inability to pay for T2D services;	**Barriers:** Less attention given to T2D care **Facilitators:** Presence of a well-established TB control programme up to the community level	**Co-management of TB-T2D comorbidity**
Sarker, 2016 [38]	Bangladesh	Cross-sectional	TB patients	The prevalence of T2D was 12.8%; The prevalence of diabetes was higher in rural areas than urban areas among the TB patients with diabetes (58.0% VS 42.0%)	**Barriers:** Funding is a challenge for the incorporation of T2D care among individuals with active TB **Facilitators:** The large number of TB patients screened – feasibility	**Co-management of TB-T2D comorbidity**
Trinidad, 2016 [39]	Republic of the Marshall Islands	Prospective cohort	T2D patients	The observed rate of TB disease among those who completed TB screening was more than 20 times higher than that reported for the general population in 2012	**Barriers:** The tuberculin skin test (TST) does not perform well in a patient with active TB disease and can miss up to 30% of prevalent cases **Facilitators:** They used TST which is the only currently available test for the diagnosis of latent TB	**Feasibility of screening for TB among T2D patients**
Fwoloshi, 2018 [40]	Zambia	Cross-sectional	TB patients	Only 4.7% of individuals with TB were found to have T2D—lower than the reported prevalence of T2D in similar cohorts of TB patients in sub-Saharan Africa but similar to the estimated prevalence of T2D in Lusaka	**Barriers:** it is not known whether the newly diagnosed T2D study participants merely had transient hyperglycemia or whether it was type 1 and not T2D **Facilitators:** screening implemented using existing resources and staff	**Feasibility of screening for T2D among TB patients**
Ncube, 2019 [41]	Zimbabwe	Cross-sectional	TB patients	TB case load (low TB notifying sites) were likely to screen more patients for T2D; Screening increased gradually per quarter over the study period; There were, however, notable losses along the screening cascade	**Barriers:** There were notable losses along the screening cascade, the reasons for which will need to be explored in future studies **Facilitators:** It was carried out in a programme setting using routinely collected data	**Feasibility of screening for T2D among TB patients**
Asante-Poku**,** 2019 [5]	Ghana	Cross-sectional	TB patients	The prevalence of T2D was 9.4%; Diabetic individuals were suggestively likely to present with TB caused by M. africanum Lineage 6 as opposed to Mycobacterium tuberculosis sensu stricto (Mtbss)	**Barriers:** Funding is a challenge **Facilitators:** it was possible to screen for T2D and identify mycobacterium different lineage	**Co-management of TB-T2D comorbidity**
Soe, 2020 [42]	Myanmar	Cross-sectional-	TB patients	Data from the TB–T2D bi-directional shows that there are several gaps in screening and linkage to care	**Barriers:** Non-screening and suboptimal screening in certain townships **Facilitators:** The study was done using data collected under routine programmatic conditions	**Co-management of TB-T2D comorbidity**
Basir, 2019 [43]	Pakistan	Cross-sectional	Individuals screened for presumptive TB and T2D	The yield of pre-T2D and T2D identified in TB patients in this programme was higher (12.4%) than the T2D prevalence in the general population of Pakistan (6.9%);	**Barriers:** User-fees for the X-ray and distance to the TB centres limited the number of diabetics undergoing TB screening **Facilitators:** Screening for T2D among TB patients presented fewer operational challenges	**Co-management of TB-T2D comorbidity**
Majumdar, 2019 [44]	India	Mixed-methods	TB & T2D patients HCW	TB patients registered at tertiary and secondary health centres were more likely to be screened than primary health centres	**Barriers:** Low patient awareness, poor knowledge of guidelines, lack of staff and inadequate training were barriers to screening **Facilitators:** The positive attitude of healthcare providers and programme staff	**Co-management of TB-T2D comorbidity**
Segafredo, 2019 [45]	Angola	Cross-sectional	TB patients	The crude prevalence of T2D among TB patients was close to 6%, slightly higher in males (6.3%) compared to females (5.7%). Age adjusted prevalence was 8%. Impaired fasting glucose (> 6.1 to < 7.0 mmol/L) was detected in 414 patients (7%)	**Barriers**: Absence of national guidelines or protocols for the integrated diagnosis and management of TB and T2D **Facilitators:** Feasible to screen for T2D within the directly observed therapy (DOTs) centres	**Feasibility of screening for T2D (and Hypertension) among TB patients**
Ekeke, 2020 [46]	Nigeria	Cross-sectional	T2D patients	Overall prevalence of TB was 0.8% (800 per 100 000)	**Barriers**: Methods of screening, recording, and reporting T2D and TB co-morbidity in routine health care settings are not well determined **Facilitators:** The number of positive cases identified following screening, yield of TB cases and the number needed to screen to make diagnosis of a TB case were encouraging	**Feasibility of screening for TB among T2D patients**
Paul, 2020 [47]	Bangladesh	Prospective cohort	TB patients	The screening for T2D among people with symptoms of TB, was effective and applicable to an ambulatory population seeking healthcare in a mix of public and private clinics	**Facilitators:** The public–private partnership design allowed recruitment of a highly representative sample of urban dwellers in Dhaka	**Feasibility of screening for T2D among TB patients**
Hewage, 2021 [48]	Sry Lanka	Cross-sectional	T2D patients	The proportion of TB detected by active screening among all T2D clinic attendees was 0.001 (6/4548)	**Facilitators:** Authors used an algorithm designed to direct study units into different care pathways based on pathophysiological explained risk factors for TB among the T2D patients	**Feasibility of screening for TB among T2D patients**
Arini, 2022 [49]	Indonesia	Qualitative	Healthcare workers	Operational constraints in collaborative TB-T2D care and control are more prominent in TB case finding and management	**Barriers:** Poor collaboration between private and public sector in the management of TB-T2D **Facilitators:** private health facilities have the potential to conduct health promotion for TB-T2D, bi-directional screening, treatment, referral, and reporting within an adequate capacity-building programme and logistic supplies	**Co-management of TB-T2D comorbidity**
Nyirenda, 2022 [50]	Malawi	Retrospective chart review analysis	T2D & TB patients	9.4% of the screened TB patients were living with T2D which is suggesting **high prevalence of TB among T2D patients and high T2D among TB patients than in general population**; One hospital had an integrated care which has contributed health systems strengthening through capacity building by providing materials and employing of additional healthcare workers at the Integrated NCDs clinic	**Barriers:** Low screening coverage and low yields; Shortage of treatment cards; Cards with blank spaces which contributed to high proportion of missed data **Facilitators:** The introduction of the treatment cards made this study possible	**Co-management of TB-T2D comorbidity**

### Collating, summarizing, and reporting results

The results from existing studies were summarised and presented in a narrative format. Data extracted were structured based on the following outcomes: feasibility of bi-directional screening of TB and T2D, availability and readiness of TB and T2D integrated services, Co-management of TB-T2D comorbidity. The outcomes emerging were examined to determine whether or not they addressed the research question.

## Results

**Fig. 1:**
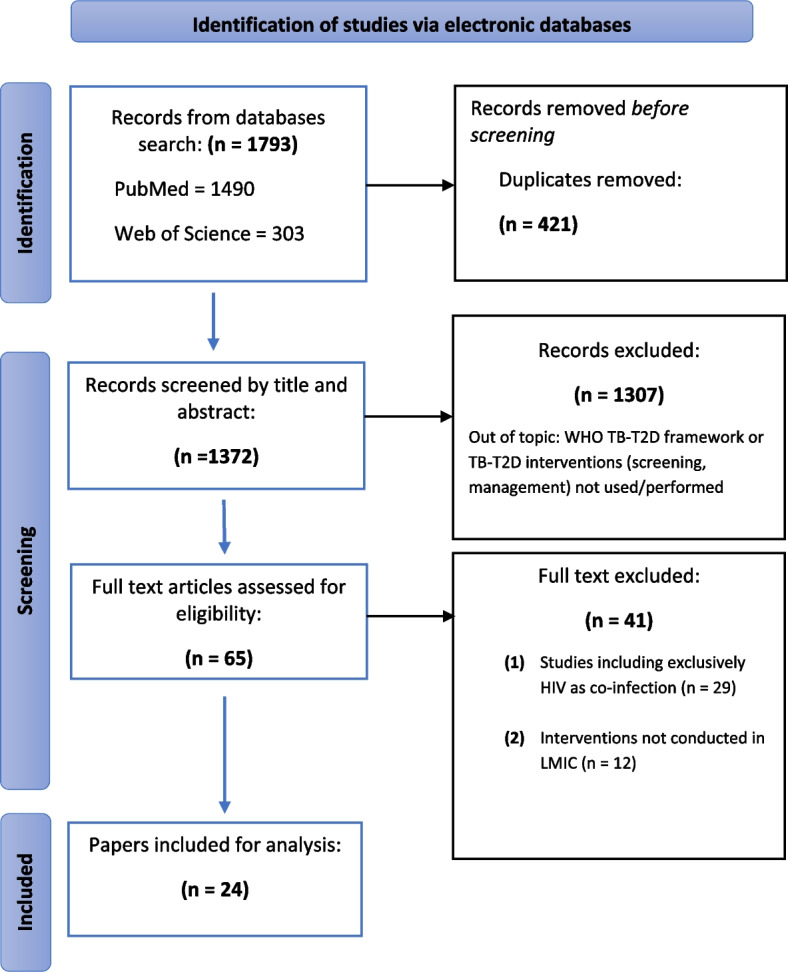
Preferred Reporting Items for Systematic Reviews and Meta-Analysis flow chart. From Moher et al.60 T2D, diabetes mellitus type 2; TB, tuberculosis

The initial search through the electronic databases, yielded a total of 1793 articles. Four hundred and twenty-one (421) articles were excluded due to duplication. Articles were screened by title and abstract and 1307 articles were excluded because they were out of topic. Finally, 65 full text articles assessed for eligibility and 41 excluded because they did not meet the inclusion criteria: 29 studies were exclusively on HIV as co-infection and 12 studies were not conducted in LMIC. Figure 1 shows the PRISMA flow chart demonstrating the screening results from each stage.

### Characteristics of included studies

A total of 24 studies [5, 29–51] reporting data from 16 countries met inclusion criteria and were included for review. The studies were divided in two groups of analysis, a) studies with TB-T2D integrated care aligned with The Union-WHO Framework and b) studies describing TB-T2D bi-directional interventions without a clear link with The Union-WHO Framework. Half of the studies were conducted in Africa (12 studies). Of which four studies related to The Union-WHO Framework category [29–32] and eight studies on bi-directional interventions [5, 36, 37, 40, 41, 45, 46, 50]. All ten studies conducted in Asia were on bi-directional interventions [33–35, 38, 42–44, 47–49], with four studies done in India (rural, urban, private and public perspectives), two studies in Bangladesh [33–35, 44] and one study in each of the following countries, Myanmar [42], Pakistan [43], Sri Lanka [48] and Indonesia [49]. Finally, only two studies conducted in Oceania [39, 51].

### Information on The Union-WHO Framework

All four paper on this topic were peer reviewed and through qualitative (2/4) and quantitative (2/4) methods (Additional file 2) [17, 29, 30, 32] showed evidence about availability, readiness, feasibility and effectiveness of TB-T2D bi-directional activities based on The Union-WHO framework recommendations. The two studies conducted in Nigeria and Tanzania followed a cross-sectional methodology (articles 1 and 2 from Table 1) and aimed at assessing the feasibility and effectiveness of screening for T2D among TB patients [29] and measuring the availability and readiness of T2D facilities to provide management for TB [30], respectively. The qualitative studies (articles 3 and 4 in Table 1below), both from Ghana, aimed at describing barriers and facilitators to the bi-directional TB-T2D screening process [31], and eliciting information on knowledge of TB-T2D comorbidity as well as systems for co-management [32]. In both studies, in-depth interviews were performed with frontline HCW implementing the TB-T2D collaborative framework [30, 31]. A description of the main findings from the review are provided below, according to the outcomes (Table 1). g.Click or tap here to enter text.

### Bi-directional interventions of TB and T2D

Seventeen studies [5, 33–39, 41–47, 51] applied quantitative methods (Table 2) to report the overlap between TB and T2D. However, two studies used qualitative approach to describe the barriers and facilitators for the bi-directional TB-T2D co-management. Only one study applied mixed methods (explanatory design) with a quantitative component (retrospective cohort), followed by a qualitative component (in-depth interviews) to describe the bi-directional TB-T2D screening services including, the enablers, barriers and solutions related to screening. Of the 17 quantitative studies, 12 are cross-sectional with seven from Asia and five from Africa. Furthermore, for are cohorts with two from Africa and one from Asia and Oceania, respectively. Finally, one study is a case–control from Oceania. From two qualitative studies, one was conducted in Africa and another in Asia. Lastly, the only study that used mixed methods approach is from Asia.

In general, the cross-sectional studies aimed at: 1) estimating the prevalence of T2D among TB patients and vice versa, 2) assessing the feasibility of TB-T2D bi-directional screening and its coverage of target groups and 3) presenting evidence for the effectiveness of bi-directional screening and management (studies 6, 7, 8, 9, 11, 12, 14, 16, 17, 19, 21 and 24 from Table 2). On the other hand, the cohort studies aimed at 1) evaluating the rate of TB in the T2D clinic, 2) evaluating the TB-T2D screening and treatment strategy and 3) determining effects of integrated care on bi-directional screening and treatment outcomes for both TB and T2D patients (studies 13, 18, 20 and 23 from Table 2). The only case–control study aimed to determine the prevalence of T2D among persons with and without TB (cases vs controls) and to determine the association between TB and T2D (study 10, Table 2). For the mixed methods study the objectives were to assess 1) the proportion of TB patients screened for T2D and vice versa, 2) factors associated with screening, and 3) the enablers, barriers and solutions related to screening (study 14, Table 2). Finally, the two qualitative studies aimed at 1) assessing the health system challenges and opportunities affecting the integration of TB-T2D services (studies 5 and 15, Table 2). A description of the main findings from the review are provided below, according to the outcomes (Table 2).

These studies showed that screening of TB patients for T2D is feasible, valuable and effective in a routine setting, predominantly, rural areas, and both in public and private clinics, resulting in earlier identification of T2D and opportunities for better management of comorbidity, and this should inform national scale-up.

## Discussion

This rapid review mapped existing literature on the implementation of The Union-WHO Framework, as well as on the bi-directional interventions of TB and T2D, in LMIC, and provided an overview of the extent of implementation from 2011 to 2022. Our review found evidence of these initiatives in 16 countries, most of them from Africa. We identified that all studies that explicitly mentioned adopting The Union-WHO framework [29–32]are from the sub-Saharan Africa region. This suggests awareness and gradual adoption of the approach proving that with an adequate reorganisation of available resources it is possible to properly implement collaborative TB and T2D activities in resource-limited settings.

Findings pointed that it is valuable, feasible and effective to implement bi-directional co-management, and this is supported by the high prevalence of TB and T2D co-existence observed in these studies, combined with the availability of a well-structured TB programme [5, 29, 33–39, 41–43, 45, 49–51]. This was congruent with the findings of Harries et al., who found that the implementation of The Union-WHO framework has the potential do stimulate and strengthen the scale-up of NCDs care and prevention programmes, which may help in reducing the global syndemic of T2D and TB.[52] This was also reported in other studies [53, 54]. However, two countries with low burden of TB and T2D did not provide evidence to support the bi-directional screening and recommend that screening should be done on a case-by-case basis [40, 48, 50]. Findings also pointed to the gaps and barriers that should be overcome to ensure a functional and effective integration of services for the co-management of TB-T2D comorbidity [37, 44, 49].

A predominant number of studies on screening for T2D among TB patients and vice versa, and co-management of TB-T2D comorbidity revealed that screening is feasible within routine practices in the healthcare system, for example using directly observed therapy (DOT) centres, resulting in earlier identification of cases and opportunities for better management of comorbidity [33, 34, 41]. This is in contrast with the findings of other studies which concluded that screening should be done on a case-by-case basis [40], especially in countries with a low burden of tuberculosis[48]. This suggests that country specific context matters and although the review intended to map evidence from LMIC, these countries do not have static and homogeneous characteristics[55].

The association between TB and T2D was identified as strong with a cross prevalence in the study groups much higher than that reported in the general population[35, 36, 38, 39, 43, 50, 51, 56], providing strong evidence that TB-T2D co-management should be prioritised. However, to ensure an efficient implementation of bi-directional activities some identified barriers should be overcome [37, 49]. This is aligned with the findings of the study by Foo et al., who found similar barriers [57]. Nevertheless, existing enablers are an important starting point [37, 49]. Other studies also demonstrated aligned facilitators, such as, the existing health systems used for TB that could be adapted to T2D management [57, 58]. Although these studies do not clearly mention that the ongoing TB-T2D bi-directional interventions were adopted from the Union-WHO Framework, its implementation is aligned with the framework’s guidelines. This may suggest that country national policy and guidelines for the co-management of TB and T2D are, indeed, based on The Union-WHO Framework whether clearly stated or not.

### Feasibility, valuable, effectiveness and acceptability

In the studies evaluated, the implementation of screening, diagnosis and management of TB and T2D was considered feasible when performed within the existing health care system, with minimum training needs however with adequate algorithm follow-up and appropriate data record and without or minimal additional resources [33–36, 40, 41, 45–48]. Additionally, findings on co-management of TB-T2D comorbidity show that patients with TB were found to have higher prevalence of T2D than reported in the general population [36, 39, 43, 50]. This representing that, these patients had the opportunity to get early diagnosis and treatment for T2D which influences the achievement of good clinical outcomes.

Thirteen studies [5, 31, 35, 36, 39–41, 43, 45, 47, 48, 50] showed high screening and diagnosis coverage and one study showed that it was possible to conduct promotion for TB-T2D care, bi-directional screening, treatment, referral, and reporting within an adequate capacity-building programme and logistic supplies [49]. Finally, from both healthcare workers and patient’s perspective, active screening strategy of TB patients for T2D was observed to be acceptable within a routine programme setting with minimal additional costs and training [5, 33–36, 38, 39, 41–43, 45–47].

#### Limitations

This rapid review had limitations worth mentioning. Firstly, only one author assessed the eligibility of studies which means a limited interpretation of findings and consequently a risk of selection bias. Secondly, it included only a peer reviewed articles published in English language, and this may have excluded some relevant information available in grey literature and studies published in other languages.

#### Recommendations

In line with other recommendations [17, 59], our findings suggest that the existence of national guidelines ensures availability and readiness of integrated services [30]. HIV-TB integration best practices should be used to help establishing and/or improving the TB-T2D collaborative interventions within a routine programme setting [33, 34, 41]. At research level, future research is needed to better understand costs, barriers, facilitators, effect of TB-T2D health programmes on clinical outcomes, clinical objectives and so, more solutions and strategies could be available to guide countries in their implementation [38, 41].

## Conclusions

This review revealed that it is valuable, feasible and effective to implement bi-directional TB and T2D activities (screening and management) according to the Union-WHO Framework recommendations, especially in countries that face TB-T2D syndemic. Additionally, it was apparent that gaps still exist in research aimed at providing evidence of costs to implement collaborative activities. There is need for TB and T2D services integration that should be done through the well-stablished TB programme. This integration of two vertical programmes, could ensure patient-centeredness, continuum of care and ultimately contribute for health systems strengthening.

### Supplementary Information

**Supplementary Material 1.**

**Supplementary Material 2.**

## Data Availability

All data generated or analysed during this study are included in this published article.

## Abbreviations

AIDS: Acquired immunodeficiency syndrome
HCW: Health care workers
HIV: Human immunodeficiency virus
LMIC: Low- and middle-income countries
NCDs: Non-communicable diseases
T2D: Diabetes Mellitus Type 2
TB: Tuberculosis
The Union: International Union Against Tuberculosis and Lung Disease
The Union-WHO Framework: The collaborative framework for care and control of TB and T2D
WHO: World Health Organization
