# Supplementary material for: The collaborative framework for the management of tuberculosis and type 2 diabetes syndemic in low- and middle-income countries: a rapid review
Source: BMC Public Health. 2024 Mar 7;24:738. doi: 10.1186/s12889-024-18256-9 (PMC10921776; doi:10.1186/s12889-024-18256-9)
Supplement: Supplementary file 1 — Supplementary Material 1. [file 12889_2024_18256_MOESM1_ESM.docx]

# Annex

1 Developing the research question using PICo approach

| ACRONYM | MEANING | ATTRIBUTE | RATIONALE |
| --- | --- | --- | --- |
| P | **P**opulation | TB patients  T2D patients  [all ages] | Population affected by both conditions and representing the syndemic burden |
| I | Phenomenon of **I**nterest | The Union-WHO Framework Interventions | Collaborative framework being a relatively new approach in the co-management of TB and T2D  Bi-directional screening and management so far not integrated in many LMIC |
| Co | **Co**ntext | Low- and middle-income countries (LMIC)  From the year 2011 | Countries with the highest co-burden of TB and T2D  Existing barriers and facilitators in the implementation of the framework/interventions since its launch in 2011  To map and capture recent evidence |

Based on the rationale in the table above, the study objectives were summarized with the following research question:

***What evidence is available regarding the extent of implementation and results of the collaborative framework for the efficient management of tuberculosis and diabetes amongst patients with both diseases in LMIC (countries with the highest dual burden of TB and T2D), in order to address gaps and challenges in the TB-T2D continuous and integrated care?***

2 Inclusion criteria

| INCLUSION  CRITERIA | DEFINITION | RATIONALE |
| --- | --- | --- |
| Population | TB patients  T2D patients  [all ages] | Articles focusing on TB-T2D bi-directional screening and/or management in patients of all ages. |
| Phenomenon | The Union-WHO Framework Interventions for the screening and management of both diseases  Implementation of integrated care for TB-T2D | The evidence about the association between tuberculosis and diabetes syndemic is quite new as well as the framework to tackle both diseases. The implementation of The Union-WHO Framework is still scarce which negatively influences early bi-directional diagnosis and management.  **Articles assessing qualitatively and quantitatively the need, barriers, challenges and facilitators of the TB-T2D continuous and integrated care in two categories a) articles that clearly state that the TB-T2D integrated care is aligned with The Union-WHO Framework and b) articles that describe the bi-directional TB-T2D interventions without a clear link with The Union-WHO Framework.** |
| Intervention | Interventions aiming to integrate TB-T2D health care | Articles that outline a **TB-T2D screening or management interventions amongst TB and T2D patients** will be included. |
| Type of study  [methods] | Qualitative, quantitative and mixed methods studies | Quantitative studies assessing the feasibility and effectiveness of collaborative care, with Qualitative research on perspectives of healthcare workers about knowledge of the The Union-WHO Framework, comorbidity, collaboration and workload and mixed methods research that triangulate data will be included. Reviews such as systematic, narratives, and others applicable will be included, specifying data relating to LMIC. |
| Context | Low- and middle-income countries (LMIC)  Publication period: from the year 2011 to present | Articles focusing on The Union-WHO Framework in LMIC will be selected including interventions implemented in these countries. Articles will be included from 2011 (11 years), year the framework was release. |

3 Exclusion criteria

| EXCLUSION CRITERIA | RATIONALE |
| --- | --- |
| Type 1 Diabetes | **Studies** presenting evidence of type 1 diabetes. This review only includes articles on type 2 diabetes as this type contributes 90-95% of all diabetes cases, globally, and shares socio-economic, environmental, and behavioural risk factors with TB. |
| TB-HIV | **Articles** focusing exclusively on HIV or other co-infection |
| Publication period and language | **Studies** published prior to 2011 (start of The Union-WHO Framework) and those published in other languages apart from English language |

Following the exclusion criteria described above, the categories below were developed for easy documentation and quantification of excluded studies.

4 Exclusion categories

| Exclusion category | Exclusion Title |
| --- | --- |
| Out of topic  (Complete or partial) | Does not focus on TB-T2D interventions, Type 1 diabetes, exclusively HIV or other co-infections, not conducted in LMIC |
|  | Not TB and T2D patients |
|  | Languages other than English |

5 LMIC where selected studies were conducted

| Africa  8/46 | | Asia  6/23 | | Oceania  2/6 |
| --- | --- | --- | --- | --- |
| Ghana  (F*2 & I) | Zimbabwe  I | Bangladesh  I*2 | Pakistan  I | Kiribati  I |
| Nigeria  (F & I) | Zambia  I | India  I*4 | Sri Lanka  I | Micronesia– Marshall Islands  I |
| Tanzania  (F & I) | Malawi  I | Indonesia  I | Myanmar  I |  |
| Ethiopia  I | Angola  I |  |  |  |

**F = study on The Union-WHO Framework**

**I = study on bi-directional interventions**

***n = number of studies**

6 Search string for the databases

**#PUBMED**

**# 1 (WHO collaborative framework): “**WHO collaborative framework” [MeSH Terms] OR WHO collaborative framework” [tiab] OR “screening” [tiab] OR “co-management” [tiab]

**#2 (diabetes and tuberculosis):** “diabetes-tuberculosis” [MeSH Terms] OR “diabetes-tuberculosis” [tiab] OR “TB-DM” [tiab]

**# 3** (**low- and middle-income countries)**

“Low- and middle-income countries” [MeSH Terms] OR “developing countries” [tiab] OR “Burkina Faso"[tiab] OR “Burundi” [tiab] OR "Central African Republic" [tiab] OR “Chad” [tiab] OR “Congo, Democratic Republic” [tiab] OR “Eritrea” [tiab] OR “Ethiopia” [tiab] OR “Gambia” [tiab] OR “Equatorial Guinea” [tiab] OR “Guinea-Bissau” [tiab] OR “Liberia” [tiab] OR “Madagascar” [tiab] OR “Malawi” [tiab] OR “Mali” [tiab] OR “Mozambique” [tiab] OR “Niger” [tiab] OR “Rwanda” [tiab] OR “Sierra Leone” [tiab] OR “Somalia” [tiab] OR “Sudan” [tiab] OR “South Sudan” [tiab] OR “Togo” [tiab] OR “Uganda” [tiab] OR “Afghanistan” [tiab] OR “Korea” [tiab] OR “Syrian Arab Republic” [tiab] OR “Yemen” [tiab] OR “Angola” [tiab] OR “Algeria” [tiab] OR “Benin” [tiab] OR “Cape Verde” [tiab] OR “Cameroon” [tiab] OR “Comoros” [tiab] OR “Congo, Republic” [tiab] OR “Ivory Coast” [tiab] OR “Djibouti” [tiab] OR “Egypt” [tiab] OR “Mauritania” [tiab] OR “Tunisia” [tiab] OR “Eswatini” [tiab] OR “Ghana” [tiab] OR “Kenya” [tiab] OR “Lesotho” [tiab] OR “Morocco” [tiab] OR “Nigeria” [tiab] OR “São Tomé and Principe” [tiab] OR “Senegal”[tiab] OR “Tanzania” [tiab] OR “Zambia” [tiab] OR “Zimbabwe” [tiab] OR “Bangladesh” [tiab] OR “Bhutan” [tiab] OR “Cambodia” [tiab] OR “India” [tiab] OR “Indonesia” [tiab] OR “Iran” [tiab] OR “Kyrgyz Republic” [tiab] OR “Lao PDR” [tiab] OR “Mongolia” [tiab] OR “Myanmar” [tiab] OR “Nepal” [tiab] OR “Pakistan” [tiab] OR “Philippines” [tiab] OR “Sri Lanka” [tiab] OR “Tajikistan” [tiab] OR “Timor-Leste” [tiab] OR “Uzbekistan” [tiab] OR “Vietnam” [tiab] OR “West Bank and Gaza” [tiab] OR “Belize” [tiab] OR “Bolivia” [tiab] OR “El Salvador” [tiab] OR “Haiti” [tiab] OR “Honduras” [tiab] OR “Nicaragua” [tiab] OR “Kiribati” [tiab] OR “Micronesia” [tiab] OR “Papua New Guinea” [tiab] OR “Samoa” [tiab] OR “Solomon Islands” [tiab] OR “Vanuatu” [tiab] OR “Ukraine” [tiab]

**#1 AND #2 AND #3**

**#Filter:** 01/01/2011 – 20/05/2022, English language, Humans

**#Web of Science**

**# 1 (WHO collaborative framework)**

TS=(“WHO collaborative framework” OR WHO collaborative framework” OR “screening” OR “co-management”)

**#2 (diabetes and tuberculosis)**

TS=(“diabetes-tuberculosis” OR “TB-DM”)

**# 3** (**low- and middle-income countries)**

TS=(“Low- and middle-income countries” OR “developing countries” OR “Burkina Faso" OR “Burundi” OR "Central African Republic" OR “Chad” OR “Congo, Democratic Republic” OR “Eritrea” OR “Ethiopia” OR “Gambia” OR “Equatorial Guinea” OR “Guinea-Bissau” OR “Liberia” OR “Madagascar” OR “Malawi” OR “Mali” OR “Mozambique” OR “Niger” OR “Rwanda” OR “Sierra Leone” OR “Somalia” OR “Sudan” OR “South Sudan” OR “Togo” OR “Uganda” OR “Afghanistan” OR “Korea” OR “Syrian Arab Republic” OR “Yemen” OR “Angola” OR “Algeria” OR “Benin” OR “Cape Verde” OR “Cameroon” OR “Comoros” “Congo, Republic” OR “Ivory Coast” OR “Djibouti” OR “Egypt” OR “Mauritania” OR “Tunisia” OR “Eswatini” OR “Ghana” OR “Kenya” OR “Lesotho” OR “Morocco” OR “Nigeria” OR “São Tomé and Principe” OR “Senegal” OR “Tanzania” OR “Zambia” OR “Zimbabwe” OR “Bangladesh” OR “Bhutan” OR “Cambodia” OR “India” OR “Indonesia” OR “Iran” OR “Kyrgyz Republic” OR “Lao PDR” OR “Mongolia” OR “Myanmar” OR “Nepal” OR “Pakistan” OR “Philippines” OR “Sri Lanka” OR “Tajikistan” OR “Timor-Leste” OR “Uzbekistan” OR “Vietnam” OR “West Bank and Gaza” OR “Belize” OR “Bolivia” OR “El Salvador” OR “Haiti” OR “Honduras” OR “Nicaragua” OR “Kiribati” OR “Micronesia” OR “Papua New Guinea” OR “Samoa” OR “Solomon Islands” OR “Vanuatu” OR “Ukraine”)

**#1 AND #2 AND #3**

**#Filter:** Publication year > 2011, Language: English

7 Data extraction sheet for articles retained for analysis

| Type of information | Description of data to be extracted |
| --- | --- |
| Author | Name of the first author |
| Publication year | Year of publication |
| Study design | Type of study: qualitative, quantitative, mixed methods, reviews |
| Study setting | Country, urban/rural, public/private |
| Study population | Population studied (TB and T2D patients) |
| Types of data included | Detail of data source |
| Assessed main outcomes | TB screening among T2D patients; T2D screening among TB patients; Co-management of TB-T2D comorbidity |
| Relevant findings | Brief summary of findings that contribute to answering the research question |
| Reported barriers and facilitators | Through qualitative studies from HCW perspective |
| Study limitations | As reported by the authors |
| Main conclusions | Brief summary of the conclusions |

8 Information on the Union-WHO Framework

| Author  Journal  Year | Country  Rural/urban  Public/Private | Study design | Study  population | Findings | Barriers & Facilitators | Conclusion &  Study limitations | Outcomes |
| --- | --- | --- | --- | --- | --- | --- | --- |
| 1  Ekeke at al  Nature  2017 | Nigeria  Urban  Public & private | Cross-sectional implementation study – under programme conditions | 2094 TB patients | T2D screening was conducted in large public and private facilities across Southern Nigeria under programme conditions with minimal additional training to the HCW; T2D prevalence was 9.4%; Factors associated with T2D were: age > 40 years old, rural residence and private health facility care. | **Facilitators:** Authors were able to implement the screening in a routine programme setting across multiple regions and facilities with minimal additional costs and training. | Screening can detect more than half of undiagnosed T2D among newly diagnosed TB patients; **Active screening strategy of TB patients for T2D is feasible and acceptable.**  **Limitations:** The results may not be representative of all TB treatment settings; Previously-treated TB patients excluded; anthropometric measurement not undertaken. | Feasibility and effectiveness of T2D screening among TB patients |
| 2  Shayo at al  BMC Public Health  2019 | Tanzania  Rural & Urban  Public & Private | Cross-sectional - Secondary analysis of the 2014–2015 Tanzania Service Provision Assessment Survey (TPSA) data | 619 health facilities  67.3% Rural  66.4% Public | Only 238 (38.4%) of all T2D facilities offer diagnosis and treatment for TB; The overall readiness of T2D facilities to provide TB services was low (12.6%); Public diabetes facilities had comparatively higher availability of TB services than private ones; Services available more in rural T2D facilities than in urban. | **Barriers:** Shortage of staff trained to co-manage TB in T2D care facilities; Inadequate TB management guidelines, medications, and diagnostics.  **Facilitators:** MoH has developed the guideline for TB-T2D collaborative care; NCDs strategic plan II prioritises to train healthcare providers on the collaborative TB-T2D care. | Given the high burden of TB, T2D, and TB-T2D comorbidity **there is a need to scale-up the availability of TB services in T2D facilities.**  **Limitations:** Cross-sectional study – difficult to tell changes occurred over time; Study did not assess the availability of other important diagnostic methods for TB (culture, molecular, genome sequencing) | Availability and readiness of TB management in T2D facilities |
| 3  Salifu at al  Plos One  2020 | Ghana  Public | Exploratory Qualitative: In-depth interviews, document review and observations | 23 HCW involved in TB and T2D care | Implementing bi-directional screening was achievable, when properly implemented; Screening to detect TB among T2D patients was more organised and focused; TB task-shifting officers improved T2D patients screening for TB | **Barriers:** Delays in screening; Fear and stigmatisation of TB; Poor collaboration between TB and T2D units; Skewed funding for screening.  **Facilitators:** Increase in staff capacity; Institutionalisation of bidirectional screening. | The implementation of bi-directional screening at public health facilities **increased staff capacity, funding and institutionalisation enhanced the policy implementation process**; The screening of TB patients for T2D is yet to be prioritised, and emphasis should be put on the design for cost-effective screening approaches for LMIC.  **Limitations:** Patients’ viewpoint missing; Study only includes 3 of several facilities in Northern Region; Study design did not allow for estimating prevalence. | Co-management of TB-T2D comorbidity |
| 4  Salifu at al  BMC Health Services Research  2021 | Ghana  Public | Exploratory Qualitative: semi-structured interviews, in-depth interviews. | 23 frontline HCW in implementing the TB-T2D collaborative framework | The study revealed 3 major themes: (1) Prioritisation of TB/HIV co-infection while negating TB-T2D comorbidity, (2) Poor working conditions, and (3) Coping mechanisms & 5 sub-themes: (1) Low knowledge and awareness on TB-T2D comorbidity, (2) Limited awareness of The Union-WHO framework among the HCW, (3) High workload in TB & T2D clinics, (4) Multiple roles, (5) Inadequate training and (6) Space shortage  TB HCW knew more about the policy than T2D HCW | **Barriers:** Prioritisation of TB/HIV co-infection while negating TB-T2D comorbidity, Poor working conditions, Low knowledge and awareness on TB-T2D comorbidity, Limited awareness of The Union-WHO framework among the HCW, High workload in TB & T2D clinics, Multiple roles, Inadequate training and Space shortage.  **Facilitators:** Coping mechanisms. | **There is need to prioritise and fund consistent in-service training programme for both TB and T2D HCW.** HIV/TB integration holds a promise that an integrated screening tool for TB and T2D, can be achieved.  **Limitations:** Non-inclusion of health facilities from the private sector and non-participation of community-based health planning services compounds (CHPS) | Co-management of TB-T2D comorbidity |

9 Bi-directional interventions of TB and T2D

| Author  Journal  Year | Country  Rural/urban  Public/Private | Study design | Study  population | Findings | Barriers & Facilitators | Conclusion &  Study limitations | Outcomes |
| --- | --- | --- | --- | --- | --- | --- | --- |
| 1  Achanta at al  Public Health Action  2013 | India  Rural  Public | Cross-sectional | All TB patients registered in the TB unit at the 10 Peripherals facilities | Of 381 TB patients, 374 (98%) were assessed for T2D and 19 (5.1%) were found to have the disease; Screening of TB patients for T2D can be effectively implemented within the existing framework of health care delivery;  Age was a factor significantly associated with the prevalence of T2D, and if resources are limited, it might be worth focusing the screening of TB patients on those aged ⩾40 years. | **Facilitators:** The study was **implemented without any additional resources within the existing health care system and with minimum training needs**; Screening of patients was well accepted in the community; | **Screening of TB patients for T2D was feasible and effective**, and this should inform national scale-up; It is acknowledged worldwide that timely referral of cases for proper care and management is vital in co-morbid conditions and the co-location and integration of services is key to successful programme collaboration.  **Limitations:** Decisions on the diagnosis of T2D were based on capillary blood glucose levels, as venous blood glucose measurements were not available. | Feasibility of screening for T2D among TB patients |
| 2  Dave at al  Public Health Action  2013 | India  85% Rural  15% Urban | Cross-sectional | All TB patients (adults and children) | At 6.5%, the prevalence of T2D in TB patients was low compared with other pilot sites in India;  Age ⩾35 years was associated with T2D. | **Barriers:** **there was no free supply of oral hypoglycaemic drugs, and some patients had to pay for these as out-of-pocket expenses**; It was a rural area with only one specialised doctor available to manage clinically complicated T2D-TB patients, and patients needed special counselling to ensure that they visited the specialist for regular follow-up.  **Facilitators: Screening was implemented within the routine system with existing staff;** With just one day of training, clinical and nursing staff were able to follow the diagnostic algorithm and record appropriate data; The time from the start of TB treatment to diagnosis was <1 week – short. | **It is feasible and valuable to screen patients with TB for T2D in a routine setting in a predominantly rural area**, resulting in earlier identification of T2D and opportunities for better management of comorbidity; Simple diagnostic technology should be in place at the start of screening activities, and **T2D drugs should be available free of charge for patients to avoid out-of-pocket expenses**.  **Limitations:** Diagnostic tools procured through external sources. | Feasibility of screening for T2D among TB patients |
| 3  Prakash at al  Public Health Action  2013 | India  Urban  Public | Cross-sectional | All diagnosed TB patients aged ⩾15 years  & All T2D patients aged ⩾15 years | Of 510 TB patients, 32 (6.3%) had been previously diagnosed with T2D; Screening among the remaining 478 patients yielded 15 (2.9%) with pre-diabetes and 15 (2.9%) newly diagnosed cases of T2D. A higher prevalence of T2D was found among patients aged ⩾40 years, patients with pulmonary TB and smokers; Of the 47 TB-T2D patients, 45 were enrolled in T2D care. Of 1670 T2D patients followed up in T2D clinics, 45 already had TB; Among the remaining 1625 patients screened, 152 (9%) had symptoms suggestive of TB; two of these were found to have the disease. | **Facilitators:** Bi-directional screening for TB and T2D implemented using existing resources and staff, thus indicating that this is feasible; **Low loss to follow-up due to the close proximity of the TB and T2D clinics.** | **Bi-directional screening for T2D and TB is feasible** and produces a high yield for T2D among TB patients. The yield of TB among T2D patients was low and needs future research using new, improved TB diagnostic tools.  **Limitations:** Authors were not able to ascertain whether a high fasting blood glucose in patients with TB was indicative of true T2D or of infection-induced hyperglycaemia – it requires periodic blood glucose testing; They could not use a glycosylated haemoglobin test for the diagnosis of T2D, and this might have led to an under diagnosis of pre-diabetes and T2D. | Co-management of TB-T2D comorbidity |
| 4  Mtwangambate at al  National Institute of Health  2014 | Tanzania  Rural  Public | Prospective cohort | All adults with T2D and cough | The prevalence of TB among adults with T2D was 7-fold higher than that reported in the general population. | **Barriers:** High rates of non-productive cough (15%).  **Facilitators:** Low-cost, ‘cough-triggered’ TB case-finding strategy that may serve as a reasonable first step for improving TB screening among adults with T2D in resource-limited settings. | TB is common among Tanzanian adults with T2D but TB case finding is challenging due to the high prevalence of non-productive cough. **This low-cost, ‘cough-triggered’ TB case finding strategy may serve as a reasonable first step for improving TB screening among adults with diabetes in sub-Saharan Africa.**  **Limitation:** Potential for bias leading to under diagnosis of TB. Among patients without productive cough, no tests were available to confirm the diagnosis. | Feasibility of screening for TB among T2D patients |
| 5  Viney at al  Tropical Medicine and International Health  2015 | Republic of Kiribati | Case-control | 275 TB cases and 499 controls were enrolled | The diabetes prevalence in cases (101, 37%) was significantly greater than in controls (94, 19%); Screening for diabetes in the TB clinic is a worthwhile public health intervention, provided that patients with T2D can access T2D care. |  | **There is a strong association between TB and T2D** in Kiribati and bi-directional screening should be conducted in this setting.  **Limitations:** Authors did not have access to TB culture to verify all diagnoses of TB. | Co-management of TB-T2D comorbidity |
| 6  Workneh at al  BMC Health Services Research  2016 | Ethiopia  Rural & Urban  Public & Private | Exploratory Qualitative: In-depth interviews, document review and observations | 44 participants (HCW, programme managers, other stakeholders involved in TB-T2D co-management) | Main themes identified: 1. Unavailability of system for continuity of T2D care; 2. Inadequate knowledge and skills of health workers; 3. Frequent stockouts of T2D supplies; 4. Patient’s inability to pay for T2D services; 5. Poor T2D data management; 6. Less attention given to T2D care; 7. Presence of a well-established TB control programme up to the community level; 8. High level of interest and readiness among HCWs, programme managers and leaders at different levels of the health care delivery system. | **Barriers:** **Unavailability of system for continuity of T2D care;** Inadequate knowledge and skills of HCW; Frequent stockouts of T2D supplies; Patient’s inability to pay for T2D services; Poor T2D data management; Less attention given to T2D care;  **Facilitators: Presence of a well-established TB control programme** up to the community level; High level of interest and readiness among HCW, programme managers and leaders at different levels of the health care delivery system. | Given the escalating dual burden of TB and T2D, and the current service gaps observed in the provision of services for T2D patients, **there is a need for integrating TB and T2D services for providing quality services to patients**.  **Limitations:** Authors did not conduct participant observation, document review and quantitative study to support the findings. As a result of this, they may not have fully assessed the natural setting in terms of service provision and programme management of both diseases. | Co-management of TB-T2D comorbidity |
| 7  Sarker at al  Plos One  2016 | Bangladesh  Urban & Rural | Cross-sectional (community based) | TB patients | Among the 1910 TB patients who participated in screening for T2D, 245 (12.8%) were found to have T2D and 296 (15.5%) to have pre-T2D; The prevalence of diabetes was higher in rural areas than urban areas among the TB patients with diabetes (58.0% VS 42.0%). | **Barriers: Funding is a challenge** for the incorporation of T2D care among individuals with active TB.  **Facilitators:** The large number of TB patients screened – feasibility. | Authors observed a higher prevalence of T2D and pre-T2D in TB patients among the general population which may challenge TB and T2D control; **T2D diagnosis, treatment and care should be integrated in the National TB Programme.**  **Limitations:** There is a possibility of underestimating the prevalence of T2D and pre-T2D among those who took part to measure random blood sugar (RBS) and fasting blood glucose only; Recruiting TB patients on treatment raises a possibility of overestimating the prevalence of T2D. | Co-management of TB-T2D comorbidity |
| 8  Trinidad at al  Journal of Clinical Tuberculosis and Other Mycobacterial Diseases  2016 | Republic of the Marshall Islands  Public | Prospective cohort | T2D patients | The observed rate of TB disease among those who completed TB screening in the Ebeye T2D clinic over a 2-year period was more than 20 times higher than that reported for the general population in 2012. | **Barriers:** The tuberculin skin test (TST) does not perform well in a patient with active TB disease and can miss up to 30% of prevalent cases.  **Facilitators:** They used TST which is the only currently available test for the diagnosis of latent TB. | **Screening T2D patients for TB can identify significant rates of TB infection and TB disease, and should be considered for other settings with a high background TB incidence. Limitations:** Although the TB case rate was high, the number of cases was small; There was no assessment of the degree of T2D control relative to TB screening outcomes among the patients. | Feasibility of screening for TB among T2D patients |
| 9  Fwoloshi at al  Canadian Journal of Infectious Diseases and Medical Microbiology  2018 | Zambia  Urban  Public | Cross-sectional | All adult patients with a new diagnosis of pulmonary TB | Only 4.7% of individuals with TB were found to have T2D - lower than the reported prevalence of T2D in similar cohorts of TB patients in sub-Saharan Africa but similar to the estimated prevalence of T2D in Lusaka. | **Barriers:** it is not known whether the newly diagnosed T2D study participants merely had transient hyperglycemia or whether it was type 1 and not T2D.  **Facilitators: screening implemented using existing resources and staff**. | **This study did not provide evidence to support the introduction of T2D screening for all TB patients.** Screening should continue to be done on a case-by-case basis. There is need to duplicate similar studies in the future.  **Limitations:** Results of this study must be interpreted with caution due to the lack of study power resulting from the low prevalence of T2D that they found among participants. | Feasibility of screening for T2D among TB patients |
| 10  Ncube at al  Public Health Action  2019 | Zimbabwe  Urban | Cross-sectional – routine programme data | 1305 newly registered TB patients aged >=15 years screened for T2D | TB prevalence – 8.5%; IIn this study, health facilities with a lower TB case load (low TB notifying sites) were likely to screen more patients for T2D; Screening increased gradually per quarter over the study period; There were, however, notable losses along the screening cascade. | **Barriers:** There were notable losses along the screening cascade, the reasons for which will need to be explored in future studies.  **Facilitators:** It was carried out in a programme setting using routinely collected data. | **Screening TB patients for T2D in a primary healthcare setting was found to be feasible**. The intervention was high-yielding and should be considered for phased national scale-up.  **Limitations:** T2D was diagnosed using FBG alone, without oral glucose tolerance or glycosylated haemoglobin testing - an underestimation of T2D prevalence in TB patients; Blood glucose was measured only once immediately after TB registration, without a confirmation test at the end of TB treatment - any cases of hyperglycaemia induced by transient infections? | Feasibility of screening for T2D among TB patients |
| 11  Asante-Poku  Plos One  2019 | Ghana  Urban and rural | Cross-sectional | 2990 TB patients screened for T2D | The prevalence of T2D at time of diagnosis among 2990 enrolled TB cases was 9.4%;  Diabetic individuals were suggestively likely to present with TB caused by M. africanum Lineage 6 as opposed to Mycobacterium tuberculosis sensu stricto (Mtbss) |  | The findings highlight the **importance of T2D and hence call for the need to raise awareness on routine screening of TB patients for T2D to ensure the use of appropriate treatment regimen.**  **Limitations:** initial random blood glucose was based on glucose measured by a point of care machine, with the potential inaccuracies; Inability to follow patients through the treatment | Co-management of TB-T2D comorbidity |
| 12  Soe at al  Tropical Medicine and Infectious Disease  2020 | Myanmar  Urban  Public | Cross-sectional- secondary analysis of routine programme data. | All TB patients enrolled for TB treatment and all T2D patients attending outpatient department | Nearly half of the TB patients were eligible for T2D screening. Of those eligible, only half were screened for T2D by random capillary blood and of those screened ~10% had high blood glucose values. About 4% of the T2D patients had undergone sputum smear examination and 2% had TB.  Data from the TB–T2D bi-directional shows that there are several gaps in screening and linkage to care. | **Barriers:** Non-screening and suboptimal screening in certain townships.  **Facilitators:** **The study was done using data collected under routine programmatic conditions (90%).** Therefore, it reflects ground level reality. | Only half of the eligible TB patients were screened for T2D and the yield of TB cases among screened T2D patients was high. **There is an urgent need for improving and scaling up bi-directional screening in the country. Limitations:** They used data recorded in programmatic records and the results may be incorrect if there are any deficiencies or errors in recording and reporting; They did not include a qualitative study component (by interviewing healthcare providers or patients) to explain the study results. | Co-management of TB-T2D comorbidity |
| 13  Basir at al  BMC Health Services Research  2019 | Pakistan  Private | Cross-sectional | Individuals screened for presumptive TB and T2D | The yield of pre-T2D and T2D identified among individuals with TB in this programme was higher (12.4%) than the T2D prevalence in the general population of Pakistan (6.9%); | **Barriers:** User-fees for the X-ray and distance to the TB centres limited the number of diabetics undergoing TB screening.  **Facilitators:** Screening for T2D among TB patients presented fewer operational challenges in their model as patients were offered screening during the time of registration for TB treatment. | Bi-directional screening for TB and T2D which includes the integration of TB diagnostics, T2D screening and TB-T2D treatment within existing health care programmes will need to address the operational challenges identified before implementing this as a strategy in public health programmes; **The private sector can be engaged cost-effectively through trainings and incentives to help achieve increased case-detection for both TB and T2D.** | Co-management of TB-T2D comorbidity |
| 14  Majumdar at al  Public Health Action  2019 | India  Rural  Public | Mixed-methods | All registered patients with TB & patients with T2D  Qualitative: patients, Healthcare providers (HCPs) | Screening for TB among T2D patients was not implemented, despite documents indicating that it had been. Of 562 TB patients, only 137 (24%) were screened for T2D. TB patients registered at tertiary and secondary health centres were more likely to be screened than primary health centres. | **Barriers:** Low patient awareness, poor knowledge of guidelines among HCPs, lack of staff and inadequate training were barriers to screening. **Facilitators:** The positive attitude of healthcare providers and programme staff; The use of routine data from a national programme. | The implementation of bi-directional screening was poor. **Adequate staffing, regular training, continuous laboratory supplies for T2D diagnosis and widespread publicity should be ensured.**  **Limitations:** The study was conducted in only one district. | Co-management of TB-T2D comorbidity |
| 15  Segafredo at al  Plos One  2019 | Angola  Urban  Public | Cross-sectional | TB patients | The crude prevalence of T2D among TB patients was close to 6%, slightly higher in males (6.3%) compared to females (5.7%). Age adjusted prevalence was 8%. Impaired fasting glucose (>6.1 to <7.0 mmol/L) was detected in 414 patients (7%). | **Barriers:** **Absence of national guidelines or protocols for the integrated diagnosis and management of TB and T2D**; Data collection system and data quality should be strengthened. **Facilitators:** Feasible to screen for T2D within the directly observed therapy (DOTs) centres. | **It is possible to screen for NCDs within the DOTS centres.** Integration of health services for both communicable and non-communicable diseases is desirable and recommended. **Limitations:** Authors cannot consider data fully representative of the general population of the country. Rural population was excluded; Only TB patients referred to DOTS centre were invited to the screening. | Feasibility of screening for T2D (and Hypertension) among TB patients |
| 16  Ekeke at al  Via Medica  2020 | Nigeria  Public | Cross-sectional | 3 457 T2D patients screened for TB | Overall prevalence of TB was 0.8% (800 per 100 000) | **Barriers:** In Nigeria, the methods of screening, recording and reporting T2D and TB co-morbidity in routine health care settings are not well determined.  **Facilitators:** The number of positive cases identified following  screening, yield of TB cases and the number needed to screen to make diagnosis of a TB case were encouraging. | **The prevalence of TB among T2D patients was higher than in the general population**; There is need for **institute active screening** for TB among T2D patients; Active case finding in the form of screening as carried out in this study is needed to end the global TB. | Feasibility of screening for TB among T2D patients |
| 17  Paul at al  Int J Infec Dis  2020 | Bangladesh  Urban  Public-Private | Prospective cohort | TB patients | T2D was found to be more common in those diagnosed with TB compared to those who were not diagnosed with TB; **The screening for T2D among people with symptoms of TB, was effective and applicable to an ambulatory population seeking healthcare in a mix of public and private clinics.** | **Facilitators:** The public-private partnership design allowed recruitment of a highly representative sample of urban dwellers in Dhaka. | A considerable burden of T2D was found among patients accessing TB diagnostics through a public-private model in urban Bangladesh, and **T2D was associated with advanced TB disease and high rate of poor treatment outcome**; The final treatment outcome could not be verified in nearly a quarter of the entire cohort as they were not followed for the extended period of time. **Limitations:** Blood glucose level testing was voluntary for the individuals evaluated for TB, and as a result a large number of individuals did not agree to be tested. | Feasibility of screening for T2D among TB patients |
| 18  Hewage at al  Plos One  2021 | Sry Lanka  Urban  Public | Cross-sectional | T2D patients above 45 years | The proportion of TB detected by active screening among all T2D clinic attendees was 0.001 (6/4548). | **Facilitators:** They used an algorithm designed to direct study units into different care pathways based on pathophysiological explained risk factors for TB among the T2D patients. | **Active screening for TB among all T2D patients at clinic settings in Sri Lanka, a country with low burden for TB, is found to be non-effective measure to enhance TB case finding, given the very low prevalence rates of TB among T2D clinic attendees**. **Limitations**: The study was conducted only in one T2D clinic which caters for a group of patients belong to middle and lower socio-economic groups in the country. | Feasibility of screening for TB among T2D patients |
| 19  Arini at al  BMC Health Services Research  2022 | Indonesia  Urban  Private | Qualitative | Private primary care providers (PPCs) healthcare workers and General Practitioners from solo practices | Themes identified in this study: (1)health system-related barriers, (2)knowledge and perception of HCW, (3)lack of implementation of bi-directional screening, and (4)needs of multisector role; **Patients have vital roles in the success of TB-T2D care and control**. Therefore, there is need to improve patient knowledge as a prerequisite to ensure their compliance with health care requirements; **Operational constraints in collaborative TB-T2D care and control are more prominent in TB case finding and management**. | **Barriers:** PPCs have not started to be involved in collaborative TB-T2D management. PPCs lack the opportunities to play active roles in collaborative TB-T2D management due to many limitations.  **Facilitators:** PPCs have the potential to conduct health promotion for TB-T2D, bi-directional screening, treatment, referral, and reporting within an adequate capacity-building programme and logistic supplies. | Although PPCs’ involvement in implementing collaborative TB-T2D services has yet to be considered, their potential role should not be neglected. Therefore, it is essential to increase their involvement by enhancing their capacity and improving the Public-Private Mix. PPCs’ engagement should be initiated and maintained to ensure the sustainability of the programme.  **Limitations:** The data interpretation is contextualised; Additional data from observations on care delivery in PPCs, document review, and other quantitative inquiries may be needed to more comprehensively assess service provision and programme management of both diseases. | Co-management of TB-T2D comorbidity |
| 20  Nyirenda at al  BMC Infectious Diseases  2022 | Malawi  Urban  Public | Retrospective chart review analysis | T2D patients & TB patients | 9.4% of the screened TB patients were living with T2D which is suggesting **high prevalence of TB among T2D patients and high T2D among TB** **patients than in general population**; One hospital had an integrated care which has contributed health systems strengthening through capacity building by providing materials and employing of additional healthcare workers at the Integrated NCDs clinic. | **Barriers:** Low screening coverage and low yields; Shortage of treatment cards; Cards with blank spaces which contributed to high proportion of missed data.  **Facilitators:** The introduction of the treatment cards made this study possible. | **Decisions on hospitals to integrate TB-T2D should be taken considering burden of the disease, and the currently rather weak evidence of an effect on bi-directional screening coverage and treatment outcomes of both diseases, and the barriers faced in the local context as well as existing guidelines. Limitations:** High proportion of data from patients who were still on treatment were collected due to missing data of the old records, and that the improvised notebooks could not capture all needed data elements as indicated on the card. | Co-management of TB-T2D comorbidity |
